# The effect of sexually transmitted infections health education on youth knowledge and attitudes: a pre-post interventional study

**DOI:** 10.1186/s12889-025-23796-9

**Published:** 2025-09-01

**Authors:** Naglaa A. El-Sherbiny, Nashwa S. Hamed, Shaimaa Sayed Eid, Asmaa Younis Elsary

**Affiliations:** Faculty of Medicine, Fayoum University, Fayoum, Egypt

**Keywords:** Adolescents, Attitude, Knowledge, Sexually transmitted infections, Students

## Abstract

**Background:**

One of the biggest issues facing public health is sexually transmitted infections, or STIs. Every day, about a million STIs are discovered worldwide.

**Aim of the study:**

Current study aimed to evaluate how well health education sessions affected the level of improvement in STI knowledge and attitude**.**

**Methods:**

One hundred eighty-nine students participated in a Pre-Post intervention study design, 95 of whom were non-Health science and 94 of whom were health science. Data was gathered via a self-administered questionnaire. The sexually transmitted disease knowledge questionnaire (STDs-KQ), sociodemographic information, and attitudes toward STIs were also covered. Students are given access to health education programs. Prior to and twice following the intervention program, assessments of the students' knowledge and disposition were conducted.

**Results:**

The knowledge level significantly increased right after the session (p-value < 0.001). When evaluated four months later, the student's knowledge about STIs had somewhat declined from its level immediately following the intervention. Nonetheless, it is significantly higher than the pre-intervention level (*p*-value 0.001). Over the course of the study, all participants' attitude levels gradually and significantly increased (*p*-value < 0.001).After four months, the type of faculty, Maternal's educational background, and socioeconomic state were all significant predictors of the degree of knowledge. After four months, the type of collage and the level of knowledge were important predictors of the attitude level.

**Conclusion:**

The study concluded that university students' attitudes and knowledge regarding STI prevention were improved by the training sessions. Based on that conclusion all university students should get health education about STIs. Using modern technology, like as smartphone applications and online e-learning courses, could maintain and improve individuals level of knowledge about STIs. The study could raise awareness of a sensitive and very important subject that people prefer to avoid discussing.

**Supplementary Information:**

The online version contains supplementary material available at 10.1186/s12889-025-23796-9.

## Introduction

Sexually transmitted infections (STIs) or sexually transmitted diseases (STDs) are infections that are spread from one person to another by sexual contact. The contact is mainly vaginal, oral, and anal sex [1]. The transmission of more than 30 different bacteria, viruses, and parasites was known to occur during sexual contact. Eight of these are attached to the highest incidence of STIs. Of these eight diseases, four are nowadays curable: syphilis, gonorrhea, chlamydia, and trichomoniasis. The other 4 lead to chronic incurable infections: hepatitis B, herpes simplex virus (HSV or herpes), HIV, and human papillomavirus (HPV). Symptoms or diseases due to an incurable infection can be reduced or reversed with treatment [2]. 

Since their inception, sexually transmitted infections have been acknowledged as a significant public health issue, particularly in underdeveloped nations [3]. Every day, sexually transmitted infections affect more than 1 million people globally [4]. The epidemiology of STIs is not known in deaeloping nations for many explanations including poverty, cultural and social (discrimination) and inadequate diagnostic tools [5]. 

STIs represent a significant economic, social, and health burden in Egypt. 3.0% of married women in Egypt between the ages of 15 and 49 have a sexually transmitted disease [6]. 

In addition to the effects of the disease itself, STIs can have dangerous side effects. For instance, the transfer from mother to child of STIs can result in stillbirth, low birth weight, sepsis, pneumonia, newborn conjunctivitis, and birth abnormalities [7]. The second most frequent cause in stillbirths worldwide is syphilis [8]. According to evidence, genital ulcers brought on by STIs are a significant clinical symptom that can raise the risk of contracting and replicating the human immunodeficiency virus [9]. 

Risky sexual behavior refers to actions that make a person more vulnerable to problems with their sexual and reproductive health, such as sexually transmitted infections (STIs), unanticipated and unwanted pregnancies, abortion, and psychological suffering. Risky sexual behavior includes; multiple sexual partners, early sexual initiation, insecure condom use, and engaging in sex with commercial sex workers [10]. 

Youth aged between 15 and 24 years old are more likely to engage in these risky behaviors, especially in industrialized countries, which put them at a higher vulnerability to sexually transmitted illnesses. Since most young people are students, educational institutions give a suitable opportunity for putting into practice efficient strategies to diminish the burden of STIs and enhance the health of their pupils [11]. 

The World Health Organization emphasized the significance of a thorough approach to STI management in 2009, including the promotion and implementation of prevention methods, community-based interventions, trustworthy feedback-based information, and efficient clinical services for STI patients. The World Health Organization also stresses the value of education and raising public awareness since these factors motivated gradual decline in STI incidence and prevalence [12]. 

Studies examining knowledge, attitudes, and safe sex behaviors to prevent STIs are lacking, particularly for groups experiencing health disparities in women and adolescents. An interventional study on this subject has never been conducted before at Fayoum University so the aim of the work was to share in filling this knowledge gab through promoting students'knowledge and attitude toward STIs. This work contributes significantly to the ongoing dialogue around STI prevention, particularly in underdeveloped regions, and we are confident that it will be a useful resource for further studies and interventions in this area. The objective of the study is to bring attention to a topic that is both extremely significant and delicate, which is something that people typically avoid talking about as having a sexually transmitted disease can have a challenging psychological impact. Some people experience anxiety or depression. They worry about transmission to sexual partners, recurring breakouts, and trouble forming new relationships. Thankfully, many of these consequences could be reduced significantly with appropriate care and awareness of the actual dangers.

### Study objectives

The present study’s goals were to evaluate Fayoum University students' attitudes and knowledge regarding STIs. To conduct health education sessions regarding the most recent STI knowledge. To evaluate how well health education sessions affected the level of improvement in STI knowledge and attitude.

### Study hypotheses

The current study assume that health education sessions could raise university students' knowledge and attitudes toward STIs.

## Methods

### Study design and sampling

The current study was pre –post interventional study that conducted in Fayoum University. According to Epi Info 2000, a sample size of 200 students (100 health science students and 100 non- health science students) out of around 43,000 university students was calculated. A multi-stage random sample technique used in current study. Based on the expected difference in knowledge and attitude after the intervention a special formula used at a confidence interval of 95% and precision of (2%). The stratified and cluster sampling methods had been considered. Finally, the sample increased by 10% to overcome problems related to non-responses and missing data.

### Participants

The first step of the multi-stage random sample used a stratified random sample for dividing the university faculties into health science faculties (medicine- pharmacy, dentist- nurse) and non- health science faculties. In the second stage, one faculty from each stratum (the Faculty of Medicine and the Faculty of Education) selected using a cluster random sampling technique. The next step involved choosing a second-year grade at random from each faculty using a cluster random sample. To choose which students would be included, a systematic random sample was the fourth and last step. All students are included in the study after implementation of sampling technique with no exclusion criteria. From total 220 students 189 responded with a response rate of 86.7%.The study conducted for seven months from November 2021 to the end of April 2022.

### Study tool and instruments

A self-administrated questionnaire for data collection that covered the following 3themes: The first section asked about Socio-demographic variables including personal data (age, gender, college type, residence, and socioeconomic status). The Socioeconomic status measured using the updated socioeconomic status scale [13]. It included question about level of education, occupation, crowding index, income, availability of water and sewage supply at home. Various cut-off thresholds were employed to separate respondents into broad socioeconomic categories (low, medium, and high)Low socioeconomic status refers to people who scored less than 40%, while high socioeconomic status was defined as a score of over 70%, and the remainder were put in the"middle"group. The second section of the questionnaire is the sexually transmitted disease knowledge questionnaire (STDs-KQ). The STDs-KQ had 27 items. It designed to assess general knowledge about STDs and six prevalent STDs. It included questions about definition of STDs, its types, mode of transmission, and its preventive measures. The STDs-KS total score ranged from 0 to 27. Based on the sum of scores, the level of knowledge classified into three levels. Low level of knowledge considered if total score (less than 60%), moderate level knowledge (60–79%), and high level knowledge (80–100%) [14]. The third section assessed the STIs related attitudes. It included questions about students’ need to receive counseling sessions and their readiness and willingness to do screening and treatment for STIs. Following a review of the literature, the questionnaire was developed. Then, items that were particular to a culture chosen. The dermatological department reviewed it with specialists. After that, the survey translated into Arabic and pretested on a small group of students. Ten questions used to assess the participants’ attitudes toward STIs. It included their need to receive counseling sessions and their readiness and willingness to be screened and treated for STIs (all scored 1 for agree, and 0 for not sure or disagree). The scores will be classified into; positive attitude (80–100%), neutral attitude (60%−79%), and negative attitude (less than 60%) [15]. 

### Study procedure

A pilot study implemented on thirty students from both health science and non- health science backgrounds completed the preliminary data collecting form to evaluate the questionnaire's readability, comprehension, clarity, and time requirements. Consequently, the required adjustments made.

**Fig. 1:**
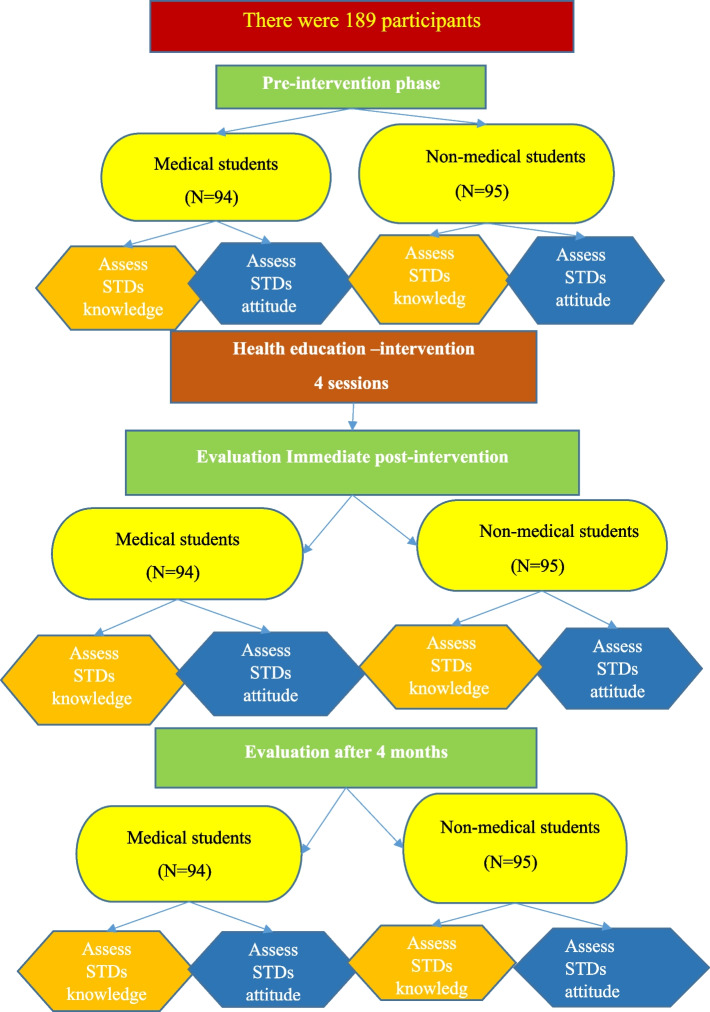
Study framework

The study was implemented on four stages: 1. The pre-intervention phase (assessment phase); at which the researchers assessed students'baseline knowledge and attitude toward sexually transmitted infections and to serve as the foundation for creating the intervention sessions. 2. The Planning phase; Based on the results obtained from the assessment phase, the researchers designed the intervention sessions and sessions’ content. Educational sessions prepared by the researchers in a form of lectures. The contents of lectures validated by experts. The sessions explained the definition, types of STIs, causes, risk factors, and routes of transmission, recognize the signs and symptoms and their consequences and determine prevention measures. 3. The intervention phase: Education sessions carried out throughout 4 sessions, with a one-week gap between each session. Each session lasted 35 to 45 min for each collage. The following were the sessions'targets: At the first session; an introduction to the educational sessions, including the purpose, significance of the topic, contents, and an explanation of essential information about STIs, such as definition, types, and the organisms that cause them as well as the mechanisms of transmission. The second and third sessions, on the other hand, were concentrated on the most significant STIs, including chlamydia, genital herpes, gonorrhea, hepatitis B, (Human immunodeficiency virus) HIV, and human papilloma virus (HPV). The fourth session focused on the importance of community and family awareness and their role in STI prevention and students completed the post-test questionnaire. Finally, we published the PowerPoint presentations on STIs on their Face book groups. 4. The Evaluation phase; the health education sessions evaluated as soon as they implemented immediately, and a follow-up evaluation conducted 4 months later. To facilitate the collection of data, we contacted the participants using their phone numbers and invited them to respond to the same tool that used in the pre-test (Fig. 1).

### Data analysis

Data analyzed using the Statistical package for social science (SPSS version 26). The mean and standard deviation computed for numerical values. For categorical variables, the number and percentage were determined, and the Chi-square test and Fisher’s exact test used for the comparison as appropriate to examine differences between observations made before, after, and at follow-up. Repeated-measures analyses of variance (ANOVAs) used to compare changes over time. A p-value less than *0.05* will consider statistically significant for all tests.

## Results

There were 189 students enrolled in current study; 94 were health science students and 95 were non- health science students. More than two third of the participants were females 133 (70.4%) and 56 (29.6%) were males with a mean age of (20.30 ± 0.81) years and most of them were single 181 (95.8%). Additionally, 99 (52.4% of them were living in rural areas.

During the pre-intervention phase, the majority of the participants, 187 (98.94%), had a low level of knowledge, with just two (1.06%) having a moderate level of knowledge. There was no statistically significant relationship with a p-value *(*> *0.05*) between students'pre-intervention level of knowledge about STIs and their socio-demographic characteristics (college, gender, residence, marital status, parents'education, parents'work, and socioeconomic status).

Immediately after the intervention, there was a statistically significant increase in knowledge level among health science single students with a higher degree of parents'education. However, no statistically significant variation in knowledge level regarding gender, residence, parents'occupation, and socioeconomic status found (Table 1).

**Table 1:** The relation between students'characteristics and level of knowledge about STIs immediately after the intervention

Students’ characteristics		Knowledge level immediately after intervention			*p*-value
		**Low (*N* = 23)**	**Moderate (*N* = 78)**	**High (*N* = 88)**	
Collage	Health science	4(17.4%)	28(35.9%)	62(70.5%)	0.001*
	Non-health science	19(82.6%)	50(64.1%)	26(29.5%)	
Gender	Female	15(65.2%)	56(71.8%)	62(70.5%)	*0.83*
	Male	8(34.8%)	22(28.2%)	26(29.5%)	
Residence	Rural	12(52.2%)	46(59%)	41(46.6%)	*0.28*
	Urban	11(47.8%)	32(41%)	47(53.4%)	
Marital status	Single	20 (87%)	73 (93.6%)	88(100%)	0.01*
	Married	3 (13%)	5 (6.4%)	0 (0%)	
Maternal education	Low	8 (34.8%)	25 (32.1%)	13 (14.8%)	0.009*
	Middle	9 (39.1%)	14 (17.9%)	25 (28.4%)	
	High	6 (26.1%)	39 (50%)	50 (56.8%)	
Father education	Low	7 (30.4%)	17 (21.8%)	8 (9.1%)	0.025*
	Middle	4 (17.4%)	20 (25.6%)	16 (18.2%)	
	High	12 (52.2%)	41 (52.6%)	64(72.7%)	
Maternal work	No	14 (60.9%)	50 (64.1%)	51 (58%)	*0.720*
	Yes	9 (39.1%)	28 (35.9%)	37 (42%)	
Father work	No	5 (21.7%)	9 (11.5%)	8 (9.1%)	*0.242*
	Yes	18 (78.3%)	69 (88.5%)	80 (90.9%)	
Socioeconomic status	Low	4 (17.4%)	9 (11.5%)	2 (2.3%)	*0.073*
	Medium	14 (60.9%)	45 (57.7%)	59 (67%)	
	High	5 (21.7%)	24 (30.8%)	27 (30.7%)	

Chi-square test

*N* Number

*Significant *p*-value < *0.05*

Four months after the intervention, there is a substantial improvement in knowledge level among health science single students (p-value *0.001*), regardless of students'gender, residence, parents'education, parents'work, or socioeconomic situation (Table 2.

**Table 2:** The relation between students'characteristics and level of knowledge about STIs four months after the intervention

Students'Characteristics		Knowledge level after 4 months (*N* = 189)			*p*-value
		**Low (*n* = 55)**	**Moderate (*n* = 93)**	**High (*n* = 41)**	
Collage	Health science	8 (14.5%)	56 (60.2%)	30 (73.2%)	< 0.001*
	Non-health science	47 (85.5%)	37 (39.8%)	11 (26.8%)	
Gender	Female	41 (74.5%)	63 (67.7%)	29 (70.7%)	*0.680*
	male	14 (25.5%)	30 (32.3%)	12 (29.3%)	
Residence	Rural	32 (58.2%)	46 (49.5%)	21 (51.2%)	*0.582*
	Urban	23 (41.8%)	47 (50.5%)	20 (48.8%)	
Marital status	Single	48 (87.3%)	92 (98.9%)	41(100%)	< 0.001 *
	Married	7 (12.7%)	1 (1.1%)	0 (0%)	
Maternal education	Low	19 (34.5%)	20 (21.5%)	7 (17.1%)	*0.119*
	Middle	16 (29.1%)	23 (24.7%)	9 (22%)	
	High	20 (36.4%)	50 (53.8%)	25 (61%)	
Father education	Low	15 (27.3%)	12 (12.9%)	5 (12.2%)	*0.053*
	Middle	12 (21.8%)	23 (24.7%)	5 (12.2%)	
	High	28 (50.9%)	58 (62.4%)	31 (75.6%)	
Maternal work	No	33 (60%)	57 (61.3%)	25 (61%)	*0.988*
	Yes	22 (40%)	36 (38.7%)	16 (39%)	
Father work	No	9 (16.4%)	8 (8.6%)	5 (12.2%)	*0.361*
	Yes	46 (83.6%)	85 (91.4%)	36 (87.8%)	
Socioeconomic status	Low	7 (12.7%)	7 (7.5%)	1 (2.4%)	*0.407*
	Medium	32 (58.2%)	57 (61.3%)	29 (70.7%)	
	High	16 (29.1%)	29 (31.2%)	11 (26.8%)	

Chi-Square Test

*N* Number

*Significant *p*-value < *0.05*

When assessing the pre-intervention student's attitude; the health science students 29(78.4%) show a higher percentage of positive attitude than non- health science students 8(21.6%); P-value < 0.001. Students with higher parents'education level and a higher socioeconomic status tend to have a higher positive attitude level (P-value < *0.001, 0.017,* and* 0.004*). the pre-intervention attitude level show no difference between students as regards their gender, residence, marital status, and parents'work p-value > *0.05*.

Immediately after the intervention, health science students and students with high socioeconomic status show a higher percentage of positive attitudes p-value (*0.002,* and* 0.07*). However, no statistically significant relationship was found between students'attitude level immediately after the intervention and their gender, residence, marital status, parents'education, or work (p-value > *0.05*) (Table 3).

**Table 3:** The relation between students'characteristics and attitude level about STIs immediately after the intervention

Students’ Characteristics		Attitude level immediately after intervention			*P*-value
		**Low(*N* = 23)**	**Neutral(*N* = 65)**	**High(*N* = 101)**	
Collage	Health science	7(30.4%)	28(43.1%)	59(58.4%)	0.002*
	Non-health science	16(69.6%)	37(56.9%)	42(41.6%)	
Gender	Female	14(60.9%)	52(80%)	67(66.3%)	*0.097*
	Male	9(39.1%)	13(20%)	34(33.7%)	
Residence	Rural	9 (39.1%)	33(50.8%)	57(56.4%)	*0.308*
	Urban	14(60.9%)	32(49.2%)	44(43.6%)	
Marital status	Single	22 (95.7%)	61(93.8%)	98 (97%)	*0.610*
	Married	1 (4.3%)	4 (6.2%)	3 (3%)	
Maternal education	Low	6 (26.1%)	19 (29.2%)	21 (20.8%)	*0.261*
	Middle	9 (39.1%)	16 (24.6%)	23 (22.8%)	
	High	8 (34.8%)	30 (46.2%)	57 (56.4%)	
Father education	Low	5 (21.7%)	17 (26.2%)	10 (9.9%)	*0.067*
	Middle	6 (26.1%)	13 (20%)	21 (20.8%)	
	High	12 (52.2%)	35 (53.8%)	70 (69.3%)	
Maternal work	No	12 (52.2%)	47 (72.3%)	56 (55.4%)	*0.062*
	Yes	11 (47.8%)	18 (27.7%)	45 (44.6%)	
Father work	No	5 (21.7%)	9 (13.8%)	8 (7.9%)	*0.139*
	Yes	18 (78.3%)	56 (86.2%)	93 (92.1%)	
Socioeconomic status	Low	4 (17.4%)	7 (10.8%)	4 (4%)	0.027*
	Medium	12 (52.2%)	46 (70.8%)	60 (59.4%)	
	High	7 (30.4%)	12 (18.5%)	37 (36.6%)	

Chi-square test

*N* Number

*Significant *p*-value < *0.05*

After 4 months of intervention, there is a considerable improvement in the percentage of positive attitude among health science students and students with highly educated fathers, (*P*-value < *0.001*, and *0.03*). However there was no change in the attitude level in terms of other characteristics of students such as gender, residence, marital status, parents'education, parents'work, and Socioeconomic status (p-value > *0.05*) (Table 4).

**Table 4:** The relation between students'basic characteristics and attitude level about STIs four months after the intervention

Students’ Characteristics		Attitude level of students after 4 months			*P*-value
		**Low(*N* = 16)**	**Neutral (*N* = 64)**	**High (*N* = 109)**	
Collage	Health science	2 (12.5%)	26 (40.6%)	67 (61.5%)	< 0.001*
	Non-health science	14 (87.5%)	38 (59.4%)	42 (38.5%)	
Gender	Female	11 (68.8%)	50 (78.1%)	71 (65.1%)	*0.198*
	Male	5 (31.3%)	14 (21.9%)	38 (34.9%)	
Residence	Rural	7 (43.8%)	31 (48.4%)	60 (55%)	*0.559*
	Urban	9 (56.3%)	33 (51.6%)	49 (45%)	
Marital status	Single	16 (100%)	59 (92.2%)	106 (97.2%)	*0.19*
	married	0 (0%)	5 (7.8%)	3 (2.8%)	
Maternal education	Low	4 (25%)	18 (28.1%)	24 (22%)	*0.118*
	Middle	8 (50%)	15 (23.4%)	25 (22.9%)	
	High	4 (25%)	31 (48.4%)	60 (55%)	
Father education	Low	4 (25%)	16 (25%)	12 (11%)	0.03*
	Middle	6 (37.5%)	13 (20.3%)	21 (19.3%)	
	High	6 (37.5%)	35 (54.7%)	76 (69.7%)	
Maternal work	No	9 (56.3%)	44 (68.8%)	62 (56.9%)	*0.281*
	Yes	7 (43.8%)	20 (31.3%)	47 (43.1%)	
Father work	No	4 (25%)	7 (10.9%)	11 (10.1%)	*0.216*
	Yes	12 (75%)	57 (89.1%)	98 (89.9%)	
Socioeconomic status	Low	3 (18.8%)	8 (12.5%)	4 (3.7%)	*0.06*
	Medium	10 (62.5%)	41 (64.1%)	67 (61.5%)	
	High	3 (18.8%)	15 (23.4%)	38 (34.9%)	

Chi-Square Test N = number *significant *p*-value < *0.05*

**Fig. 2:**
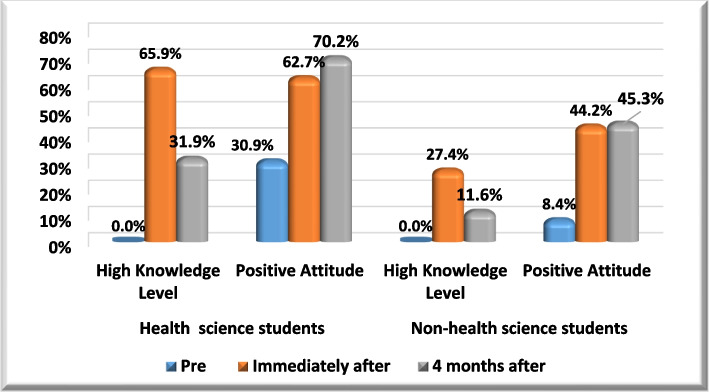
Knowledge and attitude levels follow ups in different study groups

Both health science and non-health science students show increase in knowledge and attitude levels immediately after the intervention and then show decline in their levels after four months follow up (Fig. 2).

**Fig. 3:**
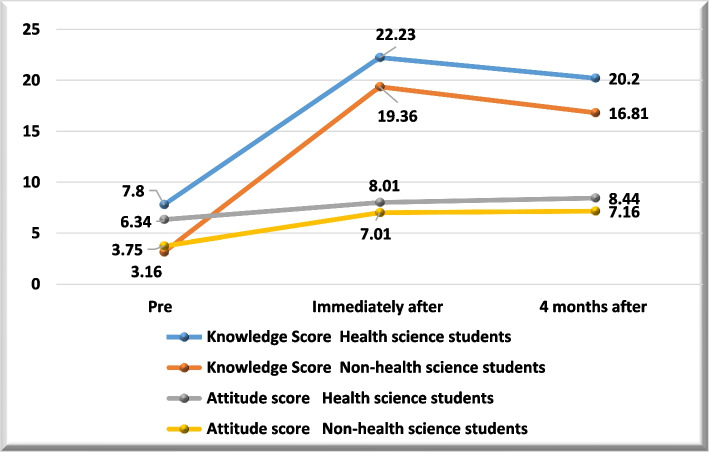
Knowledge and attitude score follow up in different study groups

Among health science students the mean knowledge score was (7.8 ± 4.4) in pre interventional stage then significantly increased to (22.23 ± 2.9) immediately after the intervention then significantly decrease to (20.2 ± 2.6) (p-value < *0.001*). Among non-health science students the mean knowledge score was (3.16 ± 3.2) in pre interventional stage then significantly increased to (19.36 ± 2.8) immediately after the intervention then significantly decrease to (16.81 ± 3.2) (p-value < *0.001*). As regards the mean attitude score among health science students; it was (6.34 ± 1.9) in pre interventional stage then significantly increased to (8.01 ± 1.6) immediately after the intervention then significantly decrease to (8.44 ± 1.5) (p-value < *0.001*). Among non-health science students the mean attitude score was (3.75 ± 2.6) in pre interventional stage then significantly increased to (7.01 ± 2.03) immediately after the intervention then significantly decrease to (7.16 ± 1.9) (p-value < *0.001*) (Fig. 3).

A multiple regression model illustrated that health science faculties, maternal education, and the socioeconomic status were predictors and risk factors that influencing knowledge level 4 months after intervention implemented. When compared to their counterparts, they elevated the risk by 3.465, 1.054, and 1.136 times with no predictive effect for other factors (Table 5).

**Table 5:** The multiple linear regression model to predict factors affecting the knowledge score four months after the intervention

Variables	Unstandardized coefficients		Standardized coefficients	t	*p*-value	95% confidence interval for B	
	**B**	**SE**	**Beta**			**Lower Bound**	**Upper Bound**
(Constant)	24.343	1.640		14.845	*0.000*	21.107	27.578
Collage(health science)	−3.465-	.462	-.514-	−7.495-	0.000*	−4.377-	−2.553-
Gender (female)	−0.793-	.471	-.107-	−1.684-	*0.094*	−1.721-	.136
Residence (rural)	0.078	.442	.012	.177	*0.860*	-.795-	.951
Maternal education (low)	1.054	.374	.257	2.815	0.005*	.315	1.793
Father education (low)	0.187	.395	.042	.473	*0.637*	-.593-	.967
Maternal work (no)	−0.605-	.546	-.088-	−1.107-	*0.270*	−1.683-	.474
Father work (no)	0.221	.701	.021	.315	*0.753*	−1.162-	1.604
Socioeconomic status (low)	−1.136-	.540	-.193-	−2.105-	0.037*	−2.202-	-.071-

*B* Beta coefficient, *Beta* standardized beta, *SE* Slandered error, *t* Test value

^*^Significant *p*-value < *0.05*

Being in a health science faculty and having a high knowledge level after four months of intervention implementation had a 0.693 and 0.552 times stronger predictive impact on attitude level four months after intervention implementation than their counterparts with no predictive effect for other factors (Table 6).

**Table 6:** The multiple linear regression model to predict factors affecting the attitude score four months after the intervention

**Variables**		**Unstandardized coefficients**		**Standardized coefficients**	**t**	***p*-value**	**95% C.I.**		
		**B**	**SE**	**Beta**			**Lower bound**	**Upper bound**	
1	(Constant)	6.390	1.145		5.581	*.000*	4.131		8.650
	Collage (health science)	−0.693-	.303	-.189-	−2.287-	0.023*	−1.292-		-.095-
	Gender (female)	0.136	.280	.034	.485	*0.628*	-.417-		.689
	Residence (ruler)	−0.252-	.263	-.069-	-.957-	*0.340*	-.772-		.267
	Maternal education (low)	−0.033-	.226	-.015-	-.146-	*0.884*	-.479-		.413
	Father education (low)	0.102	.235	.043	.436	*0.664*	-.362-		.567
	Maternal work (no)	−0.130-	.327	-.034-	-.397-	*0.692*	-.774-		.515
	Father work (no)	0.345	.417	.060	.827	*0.409*	-.478-		1.168
	Socioeconomic status (low)	0.518	.324	.162	1.598	*0.112*	-.121-		1.157
	knowledge level after 4 months (low)	0.552	.199	.213	2.773	0.006*	.159		.944

*B* Beta coefficient, *Beta* standardized beta, *SE* Slandered error, *t* Test value

*Significant p-value *<0.05*

The majority of students (93.1%) are willing to seek health science care if they experience any STI symptoms. Approximately 87.8% of students believed that STIs should be taught in schools, and 92.1% believe that being checked for STIs before getting married is critical. Only 63.5% said they would eat with an HIV-positive individual. Finally, 42.9% agreed to talk to parents about sexual matters.

## Discussion

Sexually transmitted infections (STIs) are the most pervasive and harmful infectious disorders among adolescents. According Centers for Disease Control and Prevention, (2018) found that youths aged between 15 and 24years old account for 50% of all reported STI cases. This prevalence is due to youths being more prone to participate in risky activities, specifically in developed nations that elevate their chance of acquiring STIs [16]. 

The average age of the patients in the current research was in their twenties. On the same line, the same age range was taken into account in North East Scotland and Egyptian studies looking at STIs in young people [17, 18]. 

Our study showed that 98.94% of participants had a low level of knowledge before the intervention; this may be due to talking about STIs being frowned upon in Egyptian culture due to ethical and social issues that create numerous barriers. This is consistent with a study conducted in Saudi Arabia, Egypt and India [12, 19–21]. But our result disagreed with an Iranian and an Ethiopian studies that found a total of 49% and 39.1% of the participants had low sexually transmitted infections knowledge scores respectively [22–24].

Current study showed no significant difference in the pre-intervention level of knowledge about STIs between health science and non- health science students. These findings contrast with studies conducted in Malaysian, Turkey, Saudi Arabia, and Italy that showed students from health science possessed a higher level of good knowledge of STIs than non-health sciences at pre-intervention [25–28].

In the present study, the knowledge of the students at pre-intervention was not influenced by the gender and residence of the students. This was consistent with studies conducted in Ethiopia and Iran [29, 30]. But in opposition to studies conducted in Malaysia and Iraq that detected a higher level of knowledge was observed among females and participants from the urban areas [25, 31]. This could be explained by that STIs are stigmatized and have major societal and personal consequences since they are associated with shame, embarrassment, and discrimination especially in Middle East countries.

Parents' educational backgrounds and socioeconomic status had no effect on the research group's pre-intervention knowledge level, but were connected with their level of knowledge immediately after the intervention. This matched with studies in Malaysia, Egypt and Iraq that found a high educational level and socioeconomic status mediated an increase in adolescents' awareness of STIs [31–33].

The current study found no statistically significant relationship between gender, residence, and socioeconomic status qualification concerning students'level of knowledge immediately after the intervention. This coincides with research conducted in Egypt-Banha city [18]. In contrast to another Egyptian study in Zagazig city that showed females, urban inhabitants, and high socioeconomic status had a higher post-intervention knowledge about STIs [33]. 

According to current research, knowledge and attitudes concerning STIs increased significantly, after the health education intervention was completed. This is consistent with several STI health education interventional research studies [2, 18, 34, 35]. Health education sessions were crucial in educating students about the definition, causes, modes of transmission, and prevention of STIs, which helped them develop a more positive attitude on the disease.

The results of the current study showed that students'knowledge about sexually transmitted infections was markedly significant increases immediately after the intervention then it slightly declined when tested four months later but still higher than the pre-intervention state. This is corresponding to an Egyptian conducted in Zagazig City [33] but disagreed with research in Iran and Netherlands which demonstrated that smart phone applications efficiently maintained the educational program's effects on STI-related preventative behavior [36, 37].

In terms of attitude level prior to intervention, health science students have a greater attitude level than non- health science students; this finding contradicted an Iranian research [38]. The health science students'attitude is extremely important because it has the potential to cause social stigma, lack of proper care and attention, and distance from those who have STIs. This could then result in discrimination in the provision of services for those who have these diseases.

In the present study, their gender and residents did not influence the attitude of the students at pre-intervention time. This matched with a study on health science students of Menoufia University [29]. This may be explained by the stigma associated with the diseases, which affects everyone without regard to sex or place of residence and keeps people feeling anxious and ashamed.

The educational and socioeconomic status of students'parents affected their pre-intervention attitude level in the current study. This conclusion was consistent with Ethiopian and Brazilian studies [39, 40], but not with an Egyptian study [33].

This study found a considerably marked improvement in attitudes toward STIs immediately following the intervention also slightly increased when tested four months later which is similar to Egyptian studies [2, 33]. 

The improvement in research students'attitudes immediately after the intervention compared to before the intervention seems to be brought about by their increased knowledge. In other words, having more information is linked to having a better attitude. This matched with Nigerian and Romanian studies [41, 42].

According to Becker's health belief model, people's knowledge and attitudes about health-related issues may influence how they behave in the future. This means that studing the psychoeducational implications of studies that evaluate these indices can be helpful in developing and implementing education and public health campaigns that are appropriate for the local environment [43]. 

The majority of present study students are willing to seek medical care if they experience any STI symptoms, believed that STIs should be taught in schools, and getting checked for STIs before getting married is critical, these findings were in agreement with other researches in Malaysia, Saudi Arabia, and Egypt [25, 27, 32, 33, 44]. Only 63.5% accept sharing a meal with an HIV-positive person, contrary to an Indian research that found around (84%) [45]. Indian study illustrated that 8% of study group talk to parents about sexual matters, and receive information from them. The positive relationship between parents and children was connected with reduced levels of unprotected sex, unwanted pregnancies, and STIs in teens [23]. These results support the beneficial impact of interventional orientation sessions in educating students and fostering a positive attitude on STI diseases.

### Limitation

The research topic was socially sensitive, which led to the students’ shyness. The study conducted on a certain number of university students and not all students were covered. The STI stigma prevented more students from participating. It was challenging to plan and organize four health education sessions for each study faculty. Guidance lectures were at the end of the school day, which affected the students’ ability to comprehend well due to their fatigue. Assessment challenges four months later and communication breakdowns with students to evaluate the final research evaluation. Financial support was not available and therefore a guidance booklet for students was not provided.

## Conclusion & recommendations

According to the study, the instructional sessions helped university students to develop good attitudes about STI prevention and increased their knowledge levels especially health science students. The characteristics that indicated a positive attitude after four months of the intervention were collage type and high knowledge, whereas the factors that determined the degree of knowledge were faculty type, maternal education, and socioeconomic level. Maintaining a high level of awareness could be facilitated by starting media education initiatives and making use of contemporary technologies, such as smartphone apps and online e-learning courses, or social media. Implementation of health education campaigns in schools and universities to raise youth and young adults’ awareness about STDs. Furthermore, it is essential that secondary school students take a course on sexual and reproductive health in their educational curriculums, which is a responsibility of the nation's policymakers. Further studies and researches need to be conducted to understand deeply to STDs stigma among young adults and its association with different social and cultural, religions factors.

## Supplementary Information

Supplementary Material 1.

## Data Availability

The datasets used and/or analyzed during the current study are available from the corresponding author on reasonable request.
