# Supplementary material for: The effect of sexually transmitted infections health education on youth knowledge and attitudes: a pre-post interventional study
Source: BMC Public Health. 2025 Sep 1;25:2991. doi: 10.1186/s12889-025-23796-9 (PMC12400542; doi:10.1186/s12889-025-23796-9)
Supplement: Supplementary file 1 — Supplementary Material 1. [file 12889_2025_23796_MOESM1_ESM.pdf]

The Sexually Transmitted Disease Knowledge Questionnaire
(STD-KQ; Jaworski & Carey, 2007)

Instructions: For each statement below, please circle true (T), false (F), or I don't know (DK). If you don't know, please do not guess; instead, please circle DK.

		True	False	Don't Know
1.	Genital Herpes is caused by the same virus as HIV.	T	F	DK
2.	Frequent urinary infections can cause Chlamydia.	T	F	DK
3.	There is a cure for Gonorrhea.	T	F	DK
4.	It is easier to get HIV if a person has another Sexually Transmitted Disease.	T	F	DK
5.	Human Papillomavirus (HPV) is caused by the same virus that causes HIV.	T	F	DK
6.	Having anal sex increases a person's risk of getting Hepatitis B.	T	F	DK
7.	Soon after infection with HIV a person develops open sores on his or her genitals (penis or vagina).	T	F	DK
8.	There is a cure for Chlamydia.	T	F	DK
9.	A woman who has Genital Herpes can pass the infection to her baby during childbirth.	T	F	DK
10.	A woman can look at her body and tell if she has Gonorrhea.	T	F	DK
11.	The same virus causes all of the Sexually Transmitted Diseases.	T	F	DK
12.	Human Papillomavirus (HPV) can cause Genital Warts.	T	F	DK
13.	Using a natural skin (lambskin) condom can protect a person from getting HIV.	T	F	DK
14.	Human Papillomavirus (HPV) can lead to cancer in women.	T	F	DK
15.	A man must have vaginal sex to get Genital Warts.	T	F	DK
16.	Sexually Transmitted Diseases can lead to health problems that are usually more serious for men than women.	T	F	DK
17.	A woman can tell that she has Chlamydia if she has a bad smelling odor from her vagina.	T	F	DK
18.	If a person tests positive for HIV the test can tell how sick the person will become.	T	F	DK
19.	There is a vaccine available to prevent a person from getting Gonorrhea.	T	F	DK
20.	A woman can tell by the way her body feels if she has a Sexually Transmitted Disease.	T	F	DK
21.	A person who has Genital Herpes must have open sores to give the infection to his or her sexual partner.	T	F	DK
22.	There is a vaccine that prevents a person from getting Chlamydia.	T	F	DK
23.	A man can tell by the way his body feels if he has Hepatitis B.	T	F	DK
24.	If a person had Gonorrhea in the past he or she is immune (protected) from getting it again.	T	F	DK
25.	Human Papillomavirus (HPV) can cause HIV.	T	F	DK
26.	A man can protect himself from getting Genital Warts by washing his genitals after sex.	T	F	DK
27.	There is a vaccine that can protect a person from getting Hepatitis B.	T	F	DK

Scoring for the STD Knowledge Questionnaire:

Score 1 for each correct response.

False is the correct response for these items:
1, 2, 5, 7, 10, 11, 13, 15, 16, 17, 18, 19, 20, 21, 22, 23, 24, 25, 26.

True is the correct response for the remaining items:
3, 4, 6, 8, 9, 12, 14, 27.

Total scores range from 0—27.

If you use this scale, please cite:

Jaworski, B. C., & Carey, M. P. (2007). Development and Psychometric Evaluation of a Self-administered Questionnaire to Measure Knowledge of Sexually Transmitted Diseases. *AIDS and Behavior*, 11, 557-574.

STIs related attitudes: Five questions will be used to assess the participants' attitudes toward STIs; their need to receive counseling sessions and their readiness and willingness to get screened and treated for STIs (all scored as 1 for agree, and 0 for not sure or disagree, except one question where yes scored 1, while not sure and not needed scored 0).

STIs related attitudes: Five questions will be used to assess the participants' attitudes toward STIs; their need to receive counseling sessions and their readiness and willingness to get screened and treated for STIs (all scored as 1 for agree, and 0 for not sure or disagree, except one question where yes scored 1, while not sure and not needed to score 0). the scores will be classified into; positive attitude (80-100%), neutral attitude (60%-80%) and negative attitude (less than 60%). (Bloom, 1956)

1	Both partners are responsible for prevention of transmission of STIs	Agree	not sure	disagree
2	I need counseling sessions about STIs	Yes	not sure	Not need
3	I am ready to be screened for STIs	Agree	not sure	disagree
4	I am willing to be treated for STIs if diagnosed	Agree	not sure	disagree
5	Sexual health and STIs prevention should be included in school curricula	Agree	not sure	disagree

knowledge score (STDs-KS) will be calculated by adding the number of correctly answered questions out of the 27 items Thus, the STDs-KS ranged from 0 to 27. ***(Albanghali and Othman, 2020)*** Based on the sum scores, level of knowledge will be classified into; low level knowledge (less than 60%), moderate level knowledge (60-80%) and high level knowledge (80-100%). ***(Bloom, 1956)***
